# Long-Term Benefits of Periodontal Regeneration in Severely Compromised Teeth: A Retrospective Case Series

**DOI:** 10.1155/crid/6925321

**Published:** 2025-06-16

**Authors:** James R. Collins, Nathalia Vilela, Claudio M. Pannuti

**Affiliations:** ^1^Department of Periodontology, School of Dentistry, Pontificia Universidad Católica Madre y Maestra (PUCMM), Santo Domingo, Dominican Republic; ^2^Department of Stomatology, Division of Periodontics, School of Dentistry, University of São Paulo, São Paulo, Brazil; ^3^Department of Periodontology, University of Florida College of Dentistry, Gainesville, Florida, USA

**Keywords:** case series, periodontal regeneration, periodontal surgery, tooth prognosis

## Abstract

Periodontal regeneration (PR) could represent a viable treatment alternative for severely compromised teeth. This case series presents the long-term benefits of PR with up to 13 years of follow-up. A total of six teeth in five patients were treated for intrabony defects using PR. The treatment involved the elevation of a mucoperiosteal flap, root instrumentation, and filling the bony defect with bone substitutes. Following PR, a significant reduction in probing depth (PD) was observed (mean 9.5 ± 1.87 mm; *p* = 0.03) along with a statistically significant gain in clinical attachment level (CAL) (mean 8.33 ± 1.03 mm; *p* = 0.03). Radiographic bone defect fill at the defect site was also verified (mean 9.67 ± 2.25 mm; *p* = 0.03). This case series demonstrated that PR led to reduced PD with minimal gingival recession, gain in CAL, and bone fill at defect sites, even in complex periodontal defects extending to the apical portion of the root, over a follow-up period of up to 13 years, indicating it as an effective and predictable treatment alternative.

## 1. Introduction

Periodontitis is a chronic inflammatory disease marked by attachment loss and alveolar bone resorption [1, 2]. Severely compromised teeth often exhibit intrabony defects and increased mobility, affecting their function [1, 3]. In such instances, an uncertain prognosis is established, which may lead to the recommendation of tooth extraction, followed by prosthetic rehabilitation with dental implants [4, 5].

While dental implants have demonstrated success and survival rates exceeding 95% [6–9], biological and technical complications may arise [10–12]. Complications such as mucositis and peri-implantitis have incidence rates ranging from 19% to 65% and 1% to 47%, respectively [13–15]. Further, treating these conditions is more complex and less predictable, with high recurrence rates [16, 17].

Periodontal regeneration (PR) is a viable treatment for preserving severely compromised teeth [18–20], achieving clinical improvements and long-term tooth retention in over 92% of cases treated [18, 19]. Additionally, PR can change the tooth prognosis and may be a clinically feasible and cost-effective alternative to extracting and replacing teeth with severe intrabony defects [18]. Nevertheless, personal homecare, oral health surveillance, risk factors' control (smoking cessation, hyperglycemia control, etc.), and supportive periodontal therapy (SPT) are essential to maintain clinical and radiographic long-term stability after PR [21].

Therefore, this case series is aimed at reporting the long-term benefits of PR in periodontally compromised teeth with a hopeless prognosis, underscoring PR's role as a valuable, tooth-preserving intervention in the face of periodontal disease challenges.

## 2. Case Presentation

### 2.1. Study Design and Subject Selection

**Figure 1:**
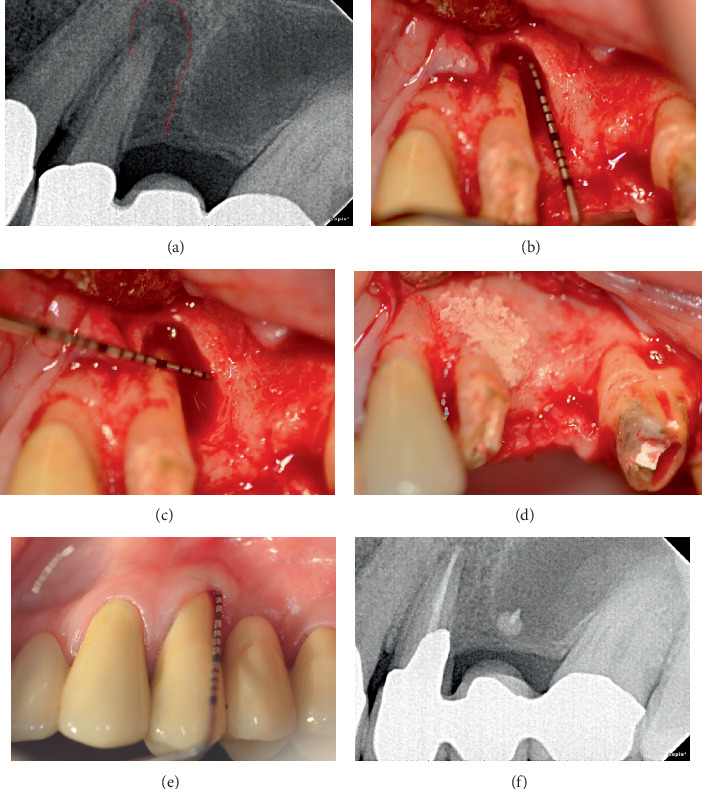
Clinical presentation of Case 1. (a) Initial periapical radiograph showing a radiolucent area in the lateral and apical third portion of the root of Tooth #24. (b, c) A two-wall bony defect was observed on the distal aspect of Tooth #24. (d) After root instrumentation, an allograft was placed followed by a collagen membrane. (e) Two years after periodontal regeneration, a 2-mm probing depth can be observed. (f) Periapical radiography with 2 years of follow-up. Complete healing of periodontal and periapical lesions can be observed.

**Figure 2:**
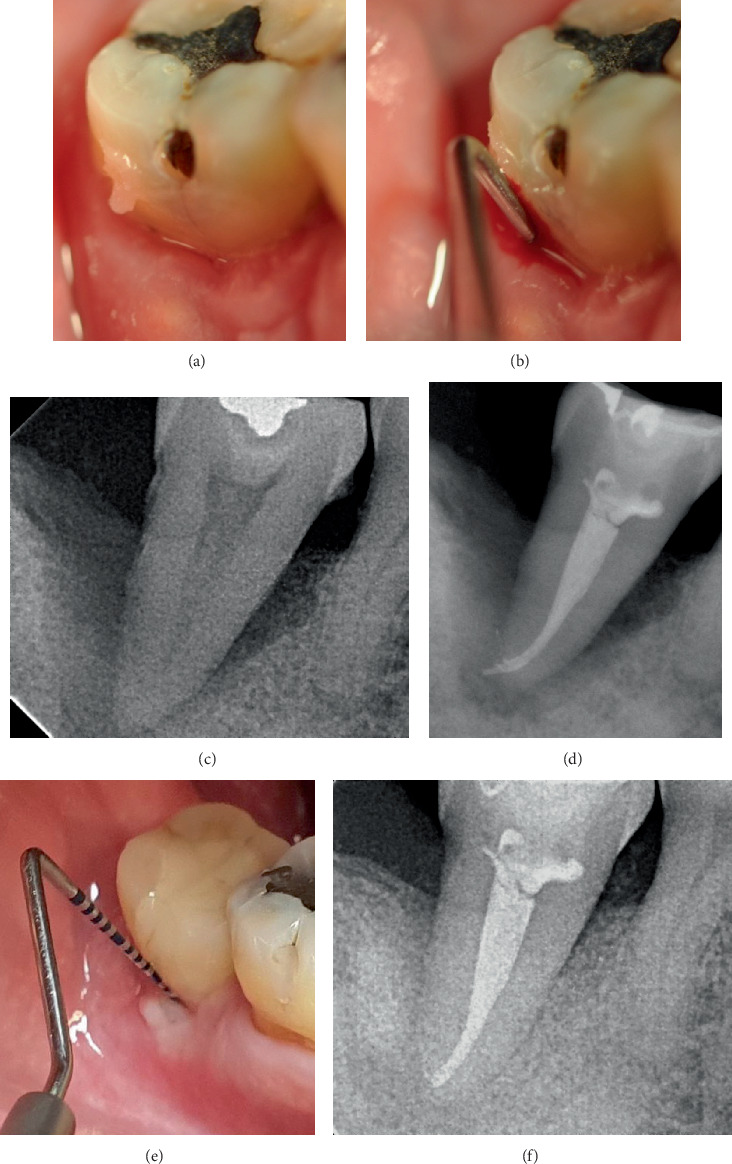
Clinical presentation of Case 2. (a) Presurgical clinical view. (b) A 15-mm periodontal pocket depth on the buccal aspect of Tooth #47. (c) Preoperative radiographic view showing severe bone loss extending to the apex of Tooth #47. (d) Root canal was performed before periodontal regeneration surgery. (e) Postoperative views at 5 years of follow-up. Probing depth was reduced to 2 mm. (f) After 5 years, an improvement in the intrabony component of the defect can be observed.

**Figure 3:**
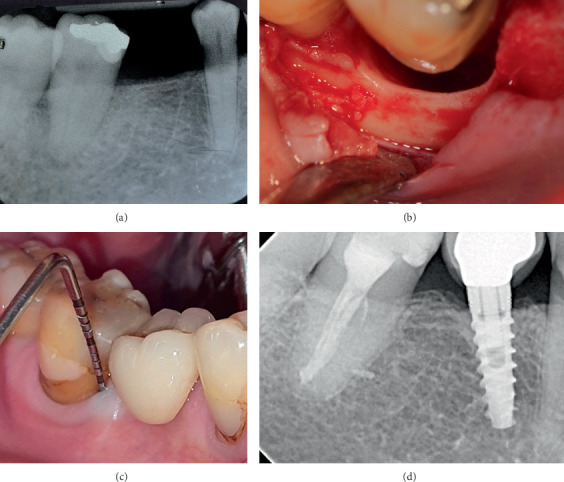
Clinical presentation of Case 3. (a) Baseline radiograph before surgery. A radiolucency at the apical–distal side of Tooth #47 can be noted. (b) A circumferential bone defect was observed on the buccal aspect of #47. (c) Thirteen-year follow-up: absence of periodontal inflammation and probing depth was reduced to 3 mm. (d) Radiograph at 13 years after surgery.

**Figure 4:**
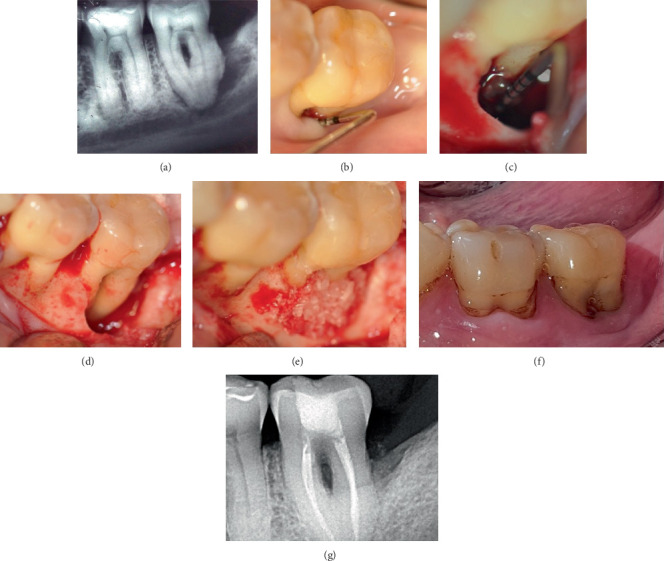
Clinical presentation of Case 4. (a) Preoperative periapical radiograph showing severe bone loss extending to the apex of Tooth #37, with a Class III furcation lesion and a short root trunk. (b) Initial probing depth, demonstrating a 14-mm periodontal pocket. (c) Approach was achieved by raising a mucoperiosteal flap, calculus was present on the root surfaces, and a large loss of periodontal support was evident. A 12-mm intrabony periodontal defect was observed. (d) Surgical root surface debridement was performed. Class III furcation involvement of the mandibular left second molar. (e) Intrabony defect was treated with a combination of enamel matrix derivative (EMD) and allograft materials. (f) Clinical view 6-year follow-up. (g) Although the furcation lesion persists, an improvement in the initial distal bone defect area can be observed.

**Figure 5:**
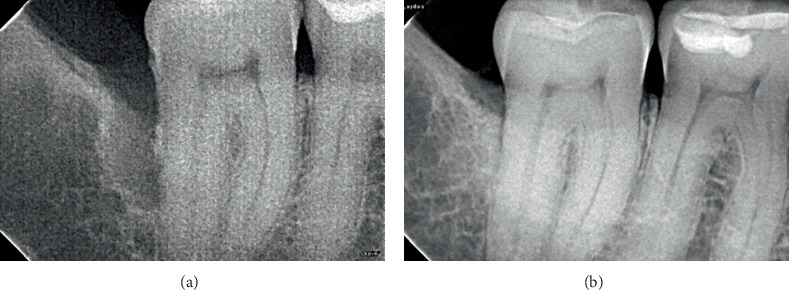
Clinical presentation of Case 5. (a) Periapical radiograph showing severe vertical bone loss extending to two-thirds of Tooth #47. (b) Six-year postoperative radiograph demonstrating improved bone support in the distal area.

**Figure 6:**
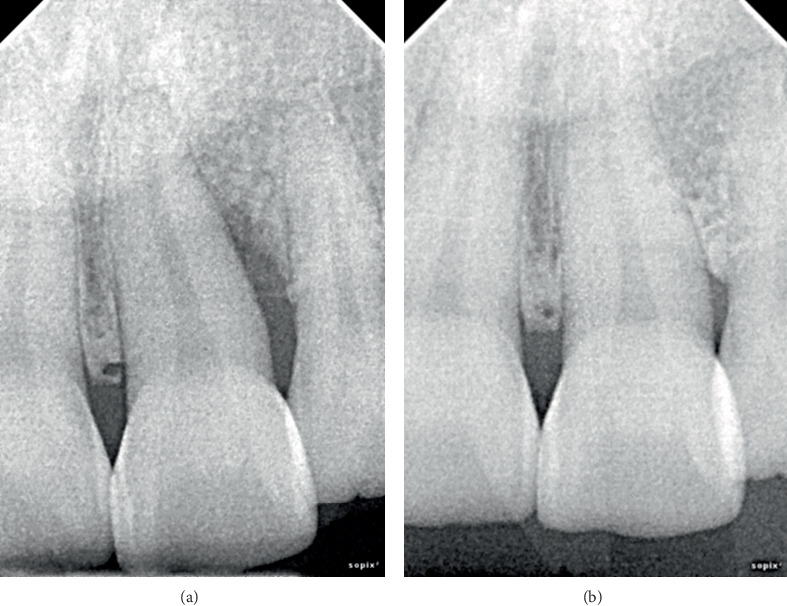
Clinical presentation of Case 6. (a) Preoperative radiograph showing an intraosseous defect on the distal side of Tooth #21. (b) Radiographic at 8 years postsurgery showing the intrabony defect filled with hard tissue.

This retrospective case series includes five nonsmoking patients (three males and two females, aged 41–64 years) who received treatment in a private practice setting between 2011 and 2024. All patients were systemically healthy (ASA I) and exhibited at least one intrabony defect involving one or two walls or a circumferential periodontal defect (Figures 1a, 1b, 1c, 2a, 2b, 2c, 2d, 3a, 3b, 4a, 4b, 4c, 4d, 5a, and 6a). The Preferred Reporting Of CasE Series in Surgery (PROCESS) guideline was adopted for reporting findings [22].

After the nonsurgical periodontal phase, all patients achieved full-mouth plaque and bleeding scores of less than 20%. Before surgery, endodontic treatment was carried out in all cases except for Cases 5 and 6 (Figures 5b and 6b), where pulp vitality was confirmed. Occlusal adjustment was performed once the occlusion was evaluated, and undesirable contacts were identified. Written informed consent was obtained from all patients.

### 2.2. Surgical Intervention

All surgeries were performed by a single, experienced operator (J.R.C.). Local anesthesia was applied using 2% lidocaine with 1:100,000 epinephrine (DFL, Rio de Janeiro). In all cases, an intrasulcular incision was made with a 15C scalpel, followed by the elevation of a mucoperiosteal flap. Careful root instrumentation was carried out using a combination of manual and ultrasonic instruments. The bone defect was filled with a bone substitute allograft in all cases, except for Case 2, where xenograft material was employed (Table 1) (Figures 1d and 4e). Root surface conditioning with 24% EDTA-Gel was performed in Cases 2, 4, and 5, and enamel matrix derivative (EMD) was placed in Case 4. In Cases 1 and 3, an allograft was placed into the bone defect and subsequently covered with a resorbable collagen membrane. The membrane was used according to the defect morphology. Specifically, it was employed in cases presenting with noncontained defects (Cases 1 and 3), while it was not used in contained defects (Cases 2, 4, 5, and 6). The selection between xenograft and allograft was based on operator preference.

Primary closure was achieved with 5-0 Vicryl absorbable sutures (Ethicon by Johnson & Johnson) using an interrupted suture technique. Postoperative indications included 875 mg of amoxicillin plus 125 mg of clavulanic acid twice daily for 5 days, 25 mg of dexketoprofen for 3 days, and local chlorhexidine spray irrigation 0.12%, three times a day for 21 days. Patients were advised to follow a soft diet and to avoid toothbrushing the surgical area for 10 days. Sutures were removed between 7 and 14 days following the surgeries. No postoperative complications were observed. Following regenerative periodontal surgery in Case 1, temporary restorations were placed which lasted 4 months. After this time, the definitive fixed restoration was started. All subjects were placed on 3-month periodontal maintenance until their final follow-ups.

### 2.3. Clinical and Radiographical Measurements

Gingival recession (GR), probing depth (PD), and clinical attachment level (CAL) at the defect sites were measured at baseline and during the follow-up visits with a PCPUNC15 periodontal probe (Hu-Friedy). Tooth mobility was assessed, and tooth prognosis was assigned according to Kwok and Caton [23] All the measurements were made by a single trained and experienced examiner (J.R.C.) at all time points. Periapical radiographs were taken before surgery and at follow-up visits using the long-cone parallel technique. Crestal bone loss (CBL) was assessed on the radiographs at defect sites, measuring from the cementoenamel junction (CEJ) to the deepest point within the intraosseous defects. Radiographic bone defect fill was determined by subtracting postsurgery CBL measurements from presurgery CBL measurements, with a positive value indicating bone fill.

### 2.4. Statistical Analysis

The individual tooth was considered the statistical unit. Descriptive data (mean and SD) were calculated. There was no normality or homoscedasticity. Thus, pre- and postsurgery measurements were compared using the Wilcoxon test. All tests were performed, considering a significance level of 5%.

## 3. Results

This case series included six cases in five patients who underwent PR of intrabony defects. No complications or adverse outcomes were observed following the surgeries.  Table 2 shows the patient's demographic data and clinical parameters before and after treatment.

A significant reduction in mean PD was observed (*p* = 0.03), from 12.17 ± 1.94 before to 2.33 ± 1.03 mm after treatment at the defect sites (median ± IQR: 12 ± 0.75 to 2 ± 0.75 mm) (Figures 1, 2, 3, and 4). There was a significant CAL improvement at the defect site from 11.33 ± 2.42 mm presurgery to 3 ± 2.45 mm postsurgery (median ± IQR: 11 ± 2.25 to 3 ± 2.25 mm) (*p* = 0.03). A significant reduction in mean CBL was observed, decreasing from 10.33 ± 2.73 mm presurgery to 0.67 ± 0.82 mm postsurgery (median ± IQR: 11.5 ± 3.25 to 0.5 ± 1 mm) (*p* = 0.03) (Table 3). Furthermore, a mean radiographic bone defect fill of 9.67 ± 2.25 (median ± IQR10.5 ± 2.5 mm) was observed at the defect sites during the 82 ± 43.21 months' follow-up period (Table 4) (Figures 1, 2, 3, 4, 5, and 6).

One-third of the cases (33.3%, Cases 2 and 3) presented tooth mobility Degree 3 before treatment. Half of the treated teeth (50%, Cases 1, 4, and 5) were assessed with tooth mobility Degree 2 prior to surgery, while only one case (16.7%, Case 6) exhibited tooth mobility Degree 1. Conversely, following PR, all treated teeth demonstrated improvements in tooth mobility. Specifically, in 83.3% of cases (Cases 1, 2, 3, 5, and 6), teeth no longer exhibited mobility; in one case (Case 4), tooth mobility decreased from Degree 2 to Degree 1. Particularly, this patient was the only one who presented a Class III furcation lesion with a buccal GR. The patient was instructed to use an oral irrigation device and interdental brushes to maintain plaque control in the furcation area. It is worth highlighting that all patients were highly motivated and committed to their periodontal support therapies and to the importance of maintaining the results obtained. The recall schedule for all patients was set at 3 months, and adjustments were made as necessary.

Four of the six treated cases initially had an unfavorable prognosis (Cases 1, 2, 3, and 4), while two (5 and 6) were considered questionable. However, all treated cases showed improvement following PR, leading to a favorable prognosis.

## 4. Discussion

This case series underscores the benefits of PR and emphasizes the importance of preserving severely compromised natural teeth. Our findings suggest that PR may be an effective long-term alternative to tooth extraction and replacement with dental implants. PR may not only halt the progression of periodontal disease but also maintain oral function and aesthetics, as supported by the outcomes observed in our cases.

Our results are in accordance with the available evidence demonstrating excellent stability and tooth retention following PR of intrabony defects [18–20, 24]. When comparing the outcomes of our cases to the literature, some similarities can be observed, such as high rates of tooth survival and significant improvement in clinical parameters. A randomized clinical trial with a 10-year follow-up demonstrated a survival rate of 88% in teeth undergoing PR, with a mean CAL gain of 7.3 ± 2.3 and residual PD of 3.4 ± 0.8 [18]. Similarly, our cases demonstrated a survival rate of 100%, with a mean CAL gain of 8.33 ± 1.03 and PD of 2.33 ± 1.03.

In this case series, both single-rooted and multirooted teeth were treated through PR, and both showed satisfactory and stable clinical and radiographic outcomes over time. This demonstrates that regardless of whether it is a single-rooted or multirooted tooth, it is possible to change the prognosis of a tooth previously considered unfavorable or questionable to a favorable prognosis without opting for irreversible treatment such as tooth extraction and implant placement.

Further, from a psychological perspective, patients generally prefer retaining their natural teeth, even hopeless teeth, if possible [18]. Preserving natural teeth enhances the quality of life by generating feelings of accomplishment, pride, a sense of control, integrity, improved oral function, greater comfort, and enhanced appearance [25]. A study comparing PR versus tooth extraction and implant placement [18] found that the PR group experienced greater improvements in oral health–related quality of life. Both groups showed improvements in treatment satisfaction, masticatory function, and aesthetics, indicating that implants did not demonstrate superiority in these outcomes.

Considering the cost associated with PR procedures, evidence suggests that despite the higher initial expense, over time, this investment demonstrates its value by promoting tooth preservation, reducing the progression of periodontitis, and decreasing expenditures related to managing periodontitis progression and tooth loss [20]. Also, when compared to dental implants, the costs associated with the placement and maintenance of implants exceeded the expenses of appropriately managing and preserving teeth affected by periodontal issues [10, 18]. Regarding patient decision-making, the majority prefer to undergo a more conservative treatment, preserving their natural teeth, rather than opting for tooth extraction, even if it entails a higher investment in treatment [26, 27].

A notable trend emerged when comparing PR to tooth extraction and dental implant placement: patients experiencing regeneration demonstrated a higher likelihood of recurrence over time. This phenomenon is expected as the decision to pursue extraction and implant placement is more radical, and complications associated with dental implants may take longer to occur [18]. Despite high survival rates, issues with dental implants can arise. Biological complications such as early failure, late failure, mucositis, and peri-implantitis may occur [28]. Managing conditions like peri-implantitis is particularly challenging, characterized by complexity and unpredictability, often leading to high recurrence rates [16, 17]. Moreover, mechanical and technical complications associated with implant-supported prostheses and their components can also arise [29, 30]. These complications, which include implant screw loosening and fracture, prosthesis fracture, and implant fracture, result in additional costs for resolution. Therefore, choosing to extract a tooth and place an implant should always be approached cautiously. This is because if an implant fails, the alternative is to place another implant, whereas if a tooth fails, there remains the option of placing an implant [31].

Regarding the selection of biomaterials, a resorbable membrane was used in cases where buccal bone wall loss required the creation of space around the diseased root surface, thereby enabling epithelial exclusion and selective repopulation by progenitor cells within the periodontal defect. Freeze-dried bone allograft (FDBA) was employed to provide an osteoconductive scaffold, support membrane positioning, and stabilize the blood clot in these noncontained defects. In contrast, due to the contained nature of the defects, membranes were not utilized in the remaining cases. Additionally, either a xenograft or an allograft was selected as the bone substitute graft based on their documented potential to stimulate PR in both preclinical and clinical studies, as no conclusive evidence favors one over the other [32].

Another biological adjunct that has gained considerable attention is the platelet-rich fibrin (PRF). However, it was not used in the present case series. Current evidence indicates that the use of PRF in combination with bone grafts for the treatment of intrabony defects may result in greater PD reduction, increased CAL gain, and enhanced radiographic bone fill [33–35]. Nonetheless, the findings from these systematic reviews revealed substantial heterogeneity among studies, which may limit the generalizability of the results [33–35]. Although PRF remains promising, further research is warranted to validate its outcomes, particularly in combination with guided tissue regeneration (GTR), emphasizing human histological assessments, patient-reported outcome measures (PROMs), and cost-effectiveness analyses [34]. Finally, while different biomaterials were employed in this study, it is important to note that case selection, comprehensive treatment planning, and precise clinical execution outweigh the significance of the biomaterial selected.

In this case series, PR proved to be an effective alternative for treating severely compromised teeth affected by intrabony defects. One strength of this study is that all surgical procedures were performed by an experienced operator using a consistent protocol. Another strength is notably the long follow-up period, achieving excellent and enduring clinical and radiographic outcomes that remained stable over up to 13 years, with no recurrence observed. Moreover, there was a clear patient preference for retaining the natural tooth. Thus, in the context of evidence-based practice, involving patients in the decision-making process is crucial. Considering their opinion and desires when determining the most suitable treatment approach is imperative for ensuring patient-centered care and predictable long-term results.

## 5. Conclusion

PR, when appropriately indicated, may be an effective alternative for preserving periodontally compromised teeth, yielding clinical and radiographic benefits, enhancing patients' quality of life, and offering favorable cost-effectiveness. Moreover, it demonstrates long-term stable outcomes.

## Figures and Tables

**Table 1:** Bone substitutes and membranes utilized in the study.

**Case**	**Bone substitute used**	**Membrane used**
1	FDBA, Puros Cancellous Particles Allograft	Biomend Extend Resorbable Collagen Membrane
2	DBBM, Geistlich Bio-Oss	No membrane employed
3	FDBA, Puros Cortical Particle Allograft	Biomend Extend Resorbable Collagen Membrane
4^a^	FDBA, Puros Cancellous Particle Allograft	No membrane employed
5^a^	FDBA, Puros Cancellous Particle Allograft	No membrane employed
6	FDBA, Puros Cortico-Cancellous Particulate Allograft	No membrane employed

Abbreviations: DBBM, deproteinized bovine bone mineral; FDBA, freeze-dried bone allograft.

^a^Cases 4 and 5 were from the same patient but involved different teeth.

**Table 2:** Patient and clinical characteristics.

			**Deepest PD at defect site (mm)**		**GM at defect site (mm)**		**CAL at defect site (mm)**		**Tooth mobility**		**Tooth prognosis**	
**Case**	**Age/sex**	**Tooth (defect site)**	**Before surgery**	**After surgery**	**Before surgery**	**After surgery**	**Before surgery**	**After surgery**	**Before surgery**	**After surgery**	**Before surgery**	**After surgery**
1	48/M	24M	13	2	2	−2	11	4	Degree 2	No mobility	U	F
2	54/M	47B	15	2	2	−1	13	3	Degree 3	No mobility	U	F
3	64/F	47M	12	2	1	−1	11	3	Degree 3	No mobility	U	F
4^a^	56/M	37B	12	4	−3	−3	15	7	Degree 2	Degree 1	U	F
5^a^	56/M	47D	12	3	2	2	10	1	Degree 2	No mobility	Q	F
6	41/F	21D	9	1	1	1	8	0	Degree 1	No mobility	Q	F
Mean ± SD			12.17 ± 1.94	2.33 ± 1.03	0.83 ± 1.94	−0.67 ± 1.86	11.33 ± 2.42	3 ± 2.45	Mean ± SD			
Median ± IQR			12 ± 0.75	2 ± 0.75	1.5 ± 1	−1 ± 2.25	11 ± 2.25	3 ± 2.25	Median ± IQR			
*p* value			0.03⁣^∗^		0.18		0.03⁣^∗^					

*Note:* All negative values for GM indicate gingival margin recession.

Abbreviations: B, buccal; CAL, clinical attachment level; D, distal; F, favorable; GM, gingival margin; IQR, interquartile range; M, mesial; PD, probing depth; Q, questionable; U, unfavorable.

^a^Cases 4 and 5 were from the same patient but involved different teeth.

⁣^∗^Significant at alpha = 5%, Wilcoxon test.

**Table 3:** Crestal bone loss before and after surgery.

**Patient**	**Tooth (defect site)**	**Follow-up (months)**	**Crestal bone loss (CBL) at defect site (mm) (BC-BD)**	
			**Before surgery**	**After surgery**
1	24M	24	12	1
2	47B	72	13	1
3	47M	156	11	0
4^a^	37B	72	12	2
5^a^	47D	72	8	0
6	21D	96	6	0
Mean ± SD		82 ± 43.21	10.33 ± 2.73	0.67 ± 0.82
Median ± IQR		72 ± 18	11.5 ± 3.25	0.5 ± 1

*Note:* BC-BD, bone crest to the base of the defect.

Abbreviations: B, buccal; D, distal; IQR, interquartile range; M, mesial.

^a^Cases 4 and 5 were from the same patient but involved different teeth.

**Table 4:** Summary of treatment outcomes.

**Patient**	**Tooth (defect site)**	**PD reduction (mm)**	**Recession increase (mm)**	**CAL gain (mm)**	**Linear bone defect fill at defect site (mm)**
1	24M	9	2	7	11
2	47B	13	1	10	12
3	47M	10	1	8	11
4^a^	37B	8	NIR	8	10
5^a^	47D	9	NR	9	8
6	21D	8	NR	8	6
Mean ± SD		9.5 ± 1.87	1.33 ± 0.58	8.33 ± 1.03	9.67 ± 2.25
Median ± IQR		9 ± 1.5	1 ± 0.5	8 ± 0.75	10.5 ± 2.5

Abbreviations: B, buccal; D, distal; IQR, interquartile range; M, mesial; NIR, no increase recession; NR, no recession.

^a^Cases 4 and 5 were from the same patient but involved different teeth.

## Data Availability

The datasets used and/or analyzed during the current study are available from the corresponding author.
